# Non-rotational medial pinning with the K-Hammer technique in pediatric supracondylar fractures: a retrospective case-control study

**DOI:** 10.3389/fped.2026.1873170

**Published:** 2026-07-09

**Authors:** Xiang Zhang, Xiaoan Bai, Changhong Li, Mi Zhou, Jiang Chen, Guanwen Sun, Fan Bai, Yijun Zhou

**Affiliations:** 1 Department of Orthopedics, Hunan Provincial People’s Hospital and The First Affiliated Hospital of Hunan Normal University, Changsha, Hunan, China; 2 Xiangya School of Medicine, Changde Hospital, Central South University (The First People’s Hospital of Changde City), Changde, Hunan, China; 3 Department of Spine Surgery, Second Xiangya Hospital of Central South University, Changsha, Hunan, China

**Keywords:** crossed pinning, K-Hammer, pediatric supracondylar humeral fracture, percutaneous pinning, ulnar nerve injury

## Abstract

**Background:**

Pediatric Gartland type III–IV extension-type supracondylar humeral fractures are associated with a risk of iatrogenic ulnar nerve injury during medial percutaneous pin insertion. The optimal technique for medial pin placement during crossed pin fixation remains debated.

**Methods:**

A retrospective case-control study was conducted involving 80 pediatric patients treated between September 2020 and September 2025. All patients had isolated closed extension-type Gartland III–IV supracondylar humeral fractures treated with two lateral and one medial crossed K-wires, with a minimum follow-up of 6 months. Patients were divided into the K-Hammer group, in which medial pinning was performed using a non-rotational K-Hammer-assisted technique with the elbow in 30°–60° extension, and the Freehand group, in which medial pinning was performed using conventional low-speed drilling with the elbow flexed at 90°. The primary outcome was symptomatic iatrogenic ulnar nerve injury within 2 weeks postoperatively. Secondary outcomes included medial pin attempts, operative time, radiographic alignment, elbow range of motion, Flynn functional grade, and postoperative complications.

**Results:**

The K-Hammer group had significantly fewer medial pin attempts [1.0 (IQR: 1.0–1.0) vs. 2.0 (IQR: 1.0–2.0), *P* < 0.001] and shorter operative time (32.8 ± 6.9 vs. 37.9 ± 9.2 min, *P* = 0.006). Intraoperative pink pulseless hand rates were comparable (10.5% vs. 4.8%, *P* = 0.416), and all patients recovered radial pulse postoperatively. Iatrogenic ulnar nerve injury occurred in 0% (0/38) of the K-Hammer group vs. 11.9% (5/42) of the Freehand group (*P* = 0.056), corresponding to an exploratory absolute risk reduction (ARR) of 11.9% and a number needed to treat (NNT) of approximately 8.4. No secondary displacement or reoperation occurred in either group. Pinsite infection and myositis ossificans rates were low and similar. At a median follow-up of 7.5 months (IQR: 6.2–11.8; range: 6–36 months), radiographic and functional were comparable between groups.

**Conclusions:**

The K-Hammer technique improves operative efficiency, reduces pin insertion attempts, and demonstrates a favorable trend toward reduction in iatrogenic ulnar nerve injury risk (ARR 11.9%, NNT = 8.4), without compromising radiographic or functional outcomes. Although statistical significance was not reached (*P* = 0.056), likely due to limited sample size, these findings suggest it is a potential technical option for medial pin placement in severe pediatric supracondylar humeral fractures, warranting confirmation in larger prospective studies.

## 1 Introduction

Supracondylar humeral fractures are the most common elbow fractures in children, accounting for more than 60% of all pediatric elbow injuries, with peak incidence at ages 5–8 years (1, 2). Gartland type III–IV fractures represent the most severe forms, carrying high risks of neurovascular injury, cubitus varus deformity, and long-term functional impairment if suboptimally managed (1, 3).

Closed reduction and percutaneous cross K-wire fixation remains one of the standard procedures for the treatment of displaced fractures, with cross-pin constructs (two lateral plus one medial K-wire) providing superior rotational and varus stability over lateral-entry-only configurations in severely unstable cases, Previous comparative studies have continued to evaluate the balance between stability and nerve injury risk in crossed vs. lateral-entry pinning constructs (4–8). However, medial pin insertion carries an inherent risk of iatrogenic ulnar nerve injury, reported in 1%–11% of cases with conventional freehand techniques (3, 6). This risk stems primarily from the use of 90° elbow flexion during medial pinning, which promotes anterior subluxation of the ulnar nerve out of the cubital tunnel—rendering it vulnerable to direct contact, laceration, or thermal injury from rotational drilling (9, 10). Ultrasonographic studies confirm that ulnar nerve instability at the pediatric elbow is present in up to 31% of children and is strongly correlated with increasing elbow flexion angle (11, 12).

Several strategies have been proposed to mitigate this risk, including mini-open nerve visualization, extension-based pinning, modified medial pin placement techniques, and ultrasound guidance (9, 13, 14). More recently, Zhou et al. introduced the K-Hammer technique: a non-rotational, mallet-assisted single-pass medial pin insertion performed with the elbow in 30°–60° extension, combining the protective effect of elbow extension on ulnar nerve position with the mechanical advantage of non-rotational penetration to minimize soft-tissue and thermal injury (15). An initial single-arm feasibility study of 34 patients reported zero iatrogenic ulnar nerve injuries; however, no controlled comparative data exist (15).

The present study addresses this evidence gap using a retrospective case-control design to compare the K-Hammer technique with conventional freehand medial pinning in 80 children with Gartland type III–IV supracondylar humeral fractures. We hypothesized that the K-Hammer strategy would be associated with fewer medial pin attempts and may show a favorable safety signal regarding iatrogenic ulnar nerve injury, while maintaining comparable radiographic and functional outcomes. Given the retrospective design, the study was intended to provide exploratory clinical evidence rather than definitive proof of superiority.

## 2 Methods

### 2.1 Study design and ethical approval

This retrospective case-control study was conducted in accordance with the STROBE (Strengthening the Reporting of Observational Studies in Epidemiology) guidelines. The study protocol was approved by the Institutional Review Board of the First People's Hospital of Changde (Approval No. YX-2023-231-01). The requirement for informed consent was waived because of the retrospective nature of the study, and all patient data were anonymized before analysis.

### 2.2 Study population and group allocation

All pediatric patients who underwent closed reduction and percutaneous crossed pinning for acute extension-type supracondylar humeral fractures (Gartland type III–IV) at our institution between September 2020 and September 2025 were retrospectively reviewed. Inclusion criteria were: (1) age 2–10 years; (2) isolated closed extension-type Gartland type III or IV supracondylar humeral fracture; (3) treatment with a crossed K-wire configuration consisting of two lateral pins and one medial pin; and (4) minimum follow-up of 6 months. Exclusion criteria included: open fractures, preoperative ulnar nerve palsy, pathological fractures, or concomitant injuries requiring surgical intervention.

Patients were divided into two groups based on the technique used for medial pin insertion:K-Hammer group: medial K-wire inserted via non-rotational, K-hammer assisted technique with the elbow maintained in 30°–60° extension; Freehand group: medial K-wire inserted using a low-speed electric drill in a blind, freehand manner with the elbow flexed at 90°.

The K-Hammer technique was introduced later during the study period and was not used fully contemporaneously with the conventional freehand technique. Therefore, potential temporal bias cannot be excluded. All procedures were performed by one of three senior pediatric orthopedic surgeons, each with more than 10 years of experience in pediatric fracture surgery.

### 2.3 Surgical techniques

All procedures were performed under general anesthesia with fluoroscopic guidance by one of three senior pediatric orthopedic surgeons. After closed reduction, two divergent lateral-entry K-wires, 1.5–2.0 mm in diameter, were inserted first to achieve provisional stability. The elbow was then positioned according to group assignment for medial pin insertion:

K-Hammer group: The elbow was extended to 30°–60°. The medial epicondyle was palpated, and thumb pressure was applied posteriorly over the medial epicondyle region to protect and displace the ulnar nerve. A K-wire was advanced perpendicularly through the medial cortex using a bone-holding forceps and a mallet without rotational drilling during cortical entry. Final transfixation through the opposite cortex was completed using brief low-speed drilling to minimize thermal injury.

Freehand group: The elbow remained flexed at 90°. The medial K-wire was inserted using a standard low-speed electric drill in a blind, freehand technique, as per conventional practice. No nerve special positioning was used.

All pins were bent and left protruding ∼1.5 cm through the skin. Postoperatively, the elbow was immobilized in a long-arm fiberglass cast at 90° flexion for 4 weeks.

### 2.4 Outcome measures

All outcome assessors were blinded to group allocation. Primary outcome:Incidence of symptomatic iatrogenic ulnar nerve injury within 2 weeks postoperatively, defined as new-onset motor and/or sensory deficit in the ulnar nerve distribution, confirmed by clinical examination. Secondary outcomes included the number of medial pin insertion attempts, total operative time, intraoperative pink pulseless hand, postoperative radial pulse recovery, radiographic alignment parameters, elbow range of motion, Flynn functional grade, radiographic union, and postoperative complications. A medial pin insertion attempt was defined as withdrawal and repositioning of the medial K-wire after initial cortical contact or penetration.

Functional outcomes at final follow-up were additionally evaluated using Flynn criteria, based on loss of carrying angle and loss of elbow motion. Outcomes were graded as excellent, good, fair, or poor according to established thresholds.

Radiographic measurements were performed independently by two pediatric orthopedic fellows using PACS software (GE Centricity). Intra-observer and inter-observer reliability were assessed using intraclass correlation coefficients (ICCs); excellent reliability was confirmed (ICC >0.85 for all angles).

### 2.5 Statistical analysis

Statistical analyses were performed using SPSS version 27.0 (IBM Corp., Armonk, NY, USA). Because this was a retrospective exploratory study, no *a priori* sample size or power calculation was performed.

Continuous variables were assessed for normality using the Shapiro–Wilk test. Normally distributed continuous variables are presented as mean ± standard deviation and were compared using the independent-samples *t*-test. Non-normally distributed continuous variables are presented as median and interquartile range and were compared using the Mann–Whitney *U*-test. Categorical variables are presented as frequencies and percentages and were compared using Pearson's chi-square test or Fisher's exact test, as appropriate. Fisher's exact test was used for rare outcomes, including iatrogenic ulnar nerve injury.

For the primary outcome, absolute risk reduction and number needed to treat were calculated as descriptive effect-size measures. Given the small number of events, these estimates were interpreted cautiously and considered exploratory rather than confirmatory. A two-sided *P* value <0.05 was considered statistically significant.

## 3 Results

A total of 80 pediatric patients met the inclusion criteria, including 38 patients in the K-Hammer group and 42 patients in the Freehand group.

Patients were divided into two groups according to the medial pin insertion technique: the K-Hammer group, in which the medial K-wire was inserted via a non-rotational, K-hammer assisted technique with the elbow maintained in 30°–60° extension; and the Freehand group, in which the medial K-wire was inserted using a low-speed electric drill in a blind, freehand manner with the elbow flexed at 90°. Of the 80 patients, 38 (47.5%) were treated in the K-Hammer group and 42 (52.5%) in the Freehand group.

### 3.1 Patient demographics and fracture characteristics

Baseline demographic and preoperative fracture characteristics are presented in Table 1. The two groups were well balanced with respect to age, sex, injured side, Gartland fracture type, time from injury to surgery, and fracture etiology, with no statistically significant intergroup differences (all *P* > 0.05).All included fractures were extension-type Gartland type III or IV injuries. No included patient had preoperative ulnar nerve palsy. All included patients completed the minimum 6-month follow-up, and no patient meeting the inclusion criteria was lost to follow-up.

**Table 1:** Comparison of patient demographics and fracture characteristics between groups.

Characteristic	Overall (*N* = 80)	K-Hammer Group (*n* = 38)	Freehand Group (*n* = 42)	*P* Value
Age, years	6.1 ± 2.8	5.9 ± 2.5	6.3 ± 3.0	0.501
Sex, male	44 (55.0%)	22 (57.9%)	22 (52.4%)	0.623
Injured side, left	41 (51.2%)	18 (47.4%)	23 (54.8%)	0.511
Gartland type IV	27 (33.8%)	11 (28.9%)	16 (38.1%)	0.382
Time to surgery, days	1.4 ± 0.7	1.3 ± 0.6	1.5 ± 0.8	0.192
Fracture etiology	–	–	–	0.837
Fall	58 (72.5%)	29 (70.7%)	29 (74.4%)	–
Traffic accident	15 (18.8%)	6 (14.6%)	9 (23.1%)	–
Fall from height	7 (8.7%)	6 (14.6%)	1 (2.6%)	–

### 3.2 Operative details and perioperative outcomes

Intraoperative and perioperative outcomes are summarized in Table 2. The median number of medial pin attempts was significantly lower in the K-Hammer group than in the Freehand group [1.0 (IQR: 1.0–1.0) vs. 2.0 (IQR: 1.0–2.0), *P* < 0.001]. Correspondingly, the mean total operative time was significantly shorter in the K-Hammer group (32.8 ± 6.9 min vs. 37.9 ± 9.2 min, *P* = 0.006). The incidence of intraoperative pink pulseless hand was comparable between groups (10.5% vs. 4.8%, *P* = 0.416), and all patients achieved full postoperative radial pulse recovery (100% in both groups).Because fluoroscopy acquisition during medial pin placement was not consistently recorded in a standardized manner, this variable was not included in the final analysis.

**Table 2:** Operative details and perioperative outcomes.

Outcome	Overall (*N* = 80)	K-Hammer Group (*n* = 38)	Freehand Group (*n* = 42)	*P* Value
Medial pin attempts	1.0 [IQR: 1.0–2.0]	1.0 [IQR: 1.0–1.0]	2.0 [IQR: 1.0–2.0]	<0.001
Total operative time, minutes	35.4 ± 8.5	32.8 ± 6.9	37.9 ± 9.2	0.006
Pink pulseless hand (intraop.)	6 (7.5%)	4 (10.5%)	2 (4.8%)	0.416
Radial pulse recovery (postop.)	80/80 (100%)	38/38 (100%)	42/42 (100%)	1.000

### 3.3 Complications and reoperations

Postoperative complication rates are detailed in Table 3. Iatrogenic ulnar nerve injury occurred in 0/38 patients (0.0%) in the K-Hammer group and 5/42 patients (11.9%) in the Freehand group (*P* = 0.056). The observed absolute risk reduction was 11.9%, corresponding to an exploratory number needed to treat of approximately 8.4. Because of the low event count and borderline *P* value, this estimate should be interpreted as a clinically interpretable signal rather than a precise or confirmatory treatment effect. The five postoperative ulnar nerve deficits in the Freehand group were identified clinically within 2 weeks after surgery and involved sensory symptoms with or without mild motor weakness in the ulnar nerve distribution. These cases were managed expectantly with clinical follow-up. No patient required secondary surgical intervention, pin removal for nerve exploration, neurolysis, or nerve reconstruction. Electromyography or nerve conduction studies were not routinely performed because the deficits showed spontaneous clinical improvement during follow-up. There were no cases of secondary displacement or reoperation in either group. Rates of superficial pin-site infection and myositis ossificans were low and similar between groups (all *P* > 0.05).

**Table 3:** Comparison of complications and reoperations.

Complication	Overall (*N* = 80)	K-Hammer Group (*n* = 38)	Freehand Group (*n* = 42)	*P* Value
Ulnar nerve injury	5 (6.3%)	0 (0.0%)	5 (11.9%)	0.056
Secondary displacement	0 (0.0%)	0 (0.0%)	0 (0.0%)	1.000
Superficial pinsite infection	3 (3.8%)	1 (2.6%)	2 (4.8%)	1.000
Myositis ossificans	3 (3.8%)	2 (5.3%)	1 (2.4%)	0.602
Reoperation	0 (0.0%)	0 (0.0%)	0 (0.0%)	1.000

Values are presented as *n* (%). Rare categorical outcomes, including iatrogenic ulnar nerve injury, were compared using Fisher's exact test. ARR and NNT were calculated as exploratory descriptive effect-size measures and should be interpreted cautiously because of the low event count.

### 3.4 Radiographic and functional outcomes

All patients completed a minimum follow-up of 6 months. Given the right-skewed distribution of follow-up duration (mean 9.7 ± 7.0 months), follow-up is more accurately characterized as a median of 7.5 months [IQR: 6.2–11.8 months; range: 6–36 months]. Follow-up duration was similar between the K-Hammer group [median 7.3 months (IQR: 6.1–11.5)] and the Freehand group [median 7.6 months (IQR: 6.3–12.1)] (*P* = 0.464). Radiographic and functional outcomes were excellent and comparable between groups at final follow-up (Table 4). No significant intergroup differences were observed in Baumann's angle, elbow flexion, elbow extension lag, carrying angle, or time to radiographic union (all *P* > 0.05). According to Flynn criteria, most patients in both groups achieved excellent or good outcomes at final follow-up, with no statistically significant difference between groups.

**Table 4:** Radiographic and functional outcomes at final follow-up.

Outcome Measure	Overall (*N* = 80)	K-Hammer Group (*n* = 38)	Freehand Group (*n* = 42)	*P* Value
Baumann's angle,°	72.1 ± 4.8	71.8 ± 4.6	72.5 ± 5.0	0.372
Elbow ROM,°				
Flexion	137.9 ± 5.3	138.4 ± 5.6	137.4 ± 5.0	0.984
Extension	−0.1 ± 3.7	−0.2 ± 3.8	−0.1 ± 3.5	0.546
Carrying angle,°	10.8 ± 4.8	10.7 ± 4.7	11.0 ± 5.0	0.825
Time to union, weeks	4.7 ± 0.8	4.6 ± 0.8	4.8 ± 0.8	0.426
Followup, months				
Median [IQR]	7.5 [6.2–11.8]	7.3 [6.1–11.5]	7.6 [6.3–12.1]	0.464
Range (min–max)	6–36	6–34	6–36	–

## 4 Discussion

The present study is a retrospective case-control study designed to provide controlled comparative evidence for the K-Hammer technique, which was first introduced and validated in a prior single-arm observational study by our group (15). The previously published article reported the feasibility and safety of the K-Hammer percutaneous fixation technique in 34 pediatric patients with extension-type supracondylar humeral fractures, demonstrating zero iatrogenic ulnar nerve injury and excellent functional outcomes. That work served as an initial proof-of-concept study to establish the technical rationale and initial safety profile of the K-Hammer method.

In contrast, the current investigation represents a controlled expansion study with a total of 80 patients, directly comparing the K-Hammer technique with conventional freehand medial pinning. The two studies are sequential, complementary, and non-duplicative: the first was a single-cohort technical introduction without a control group; the second is a case-control comparison focused on surgical efficiency, pin placement accuracy, and ulnar nerve risk reduction. No overlapping patient data, repetitive analyses, or duplicated results exist between the two publications. The present controlled study therefore addresses the critical evidence gap by providing head-to-head comparative data that were lacking in the initial report.

Pediatric extension-type supracondylar humeral fractures, especially Gartland type III–IV injuries, represent the most common elbow fractures in children and are associated with high risks of neurovascular complications and postoperative dysfunction (1, 2). Closed reduction and percutaneous cross-pinning (two lateral plus one medial K-wire) is widely used for unstable cases because of its biomechanical stability for unstable cases due to its superior biomechanical stability (4, 5). However, the risk of iatrogenic ulnar nerve injury during medial pin insertion remains a major concern, with reported incidence ranging from 1% to 11% in conventional freehand procedures (3, 16). Traditional freehand medial pinning is typically performed with the elbow in 90° flexion, a position that promotes anterior subluxation of the ulnar nerve and increases the likelihood of nerve entrapment, laceration, or thermal injury from repeated drilling (17, 18). The present study compared the safety and efficacy of the novel K-Hammer technique with the conventional freehand technique for medial K-wire placement in 80 children with displaced extension-type supracondylar fractures, providing preliminary comparative evidence regarding procedural efficiency and ulnar nerve safety.

In this study, no iatrogenic ulnar nerve injury was observed in the K-Hammer group, whereas five such injuries occurred in the Freehand group. However, this difference narrowly missed conventional statistical significance (*P* = 0.056). Therefore, the present findings should be interpreted as a hypothesis-generating safety signal rather than definitive proof that the K-Hammer technique reduces ulnar nerve injury.

The observed difference in ulnar nerve injury may be clinically relevant, but it must be interpreted cautiously. The absolute risk reduction was 11.9%, corresponding to an exploratory number needed to treat of approximately 8.4. Because of the small number of events and limited sample size, these values should not be regarded as precise estimates of treatment effect. Instead, they provide clinically interpretable effect-size measures that may be useful for designing future adequately powered prospective studies.

Importantly, the present study compared two combined procedural strategies rather than isolating a single technical variable. The K-Hammer strategy incorporated non-rotational hammer-assisted advancement, manual soft-tissue protection, and medial pin insertion with the elbow in a relatively extended position. In contrast, the conventional freehand technique was performed with the elbow flexed at approximately 90°. Given the known relationship between elbow flexion and anterior displacement or subluxation of the ulnar nerve in children, and case-based evidence that ulnar nerve instability may increase vulnerability to iatrogenic injury during medial K-wire placement, the observed reduction in nerve injury may have been driven partly, or even predominantly, by reduced elbow flexion rather than by hammer-assisted insertion itself (9, 10, 17, 19). Therefore, the relative contribution of elbow position, manual nerve protection, and non-rotational pin advancement cannot be separated in this retrospective comparison.

In terms of surgical efficiency, the K-Hammer group also demonstrated improved procedural efficiency, including fewer medial pin attempts and shorter operative time. These findings may reflect the single-pass nature of the K-Hammer strategy. However, because the K-Hammer technique was introduced later during the study period and was not used fully contemporaneously with the freehand technique, efficiency improvements may also reflect accumulated surgeon experience, workflow optimization, and evolving perioperative practice rather than the technique alone. This temporal bias is an important limitation of the present study.The one-pass characteristic of the K-Hammer strategy may reduce repeated drilling attempts and soft-tissue disturbance around the medial epicondyle (15, 16). Importantly, these efficiency improvements did not compromise fracture stability or functional outcomes. Both groups showed excellent and comparable radiographic results, including Baumann's angle, carrying angle, elbow range of motion, and time to radiographic union. No patient in either group experienced secondary displacement or required reoperation, confirming that the K-Hammer technique provides sufficient biomechanical stability for unstable supracondylar fractures, consistent with the biomechanical advantages of cross-pin constructs established in prior comparative studies (4, 5).

Although many postoperative ulnar nerve deficits after pediatric supracondylar fracture fixation are transient, persistent neurological dysfunction has been reported in referral-center and specialist peripheral nerve series (16–18, 20, 21). Such cases may require prolonged surveillance, electrophysiological assessment, neurolysis, or, rarely, reconstructive intervention (16–18). From this perspective, even a non-definitive reduction in iatrogenic ulnar nerve injury may be clinically relevant, particularly because ulnar nerve injury can impose functional, psychological, and follow-up burdens on children and their families (16–18, 20, 21). Prior specialist peripheral nerve reports have shown that persistent postoperative nerve dysfunction may occasionally result from entrapment, intraneural scarring, or severe traction injury requiring secondary intervention (16–18). Thus, strategies that may reduce the risk of iatrogenic ulnar nerve injury warrant further investigation, even when preliminary studies are underpowered for definitive statistica confirmation.

The overall complication profile further supports the clinical value of the K-Hammer technique. The incidence of pin-site infection and myositis ossificans was low and similar between groups, indicating comparable soft-tissue tolerance. Most importantly, no patient in the K-Hammer group developed ulnar nerve symptoms, whereas 5 patients in the freehand group had transient or persistent nerve dysfunction. Iatrogenic ulnar nerve injury can lead to long-term sensory-motor deficits, repeated follow-ups, and increased medical costs (20, 21). These findings suggest that further investigation of strategies to reduce iatrogenic ulnar nerve injury is warranted.

Radiographic and functional outcomes were comparable between groups. No patient in either group experienced secondary displacement or required reoperation, and final Baumann's angle, carrying angle, elbow range of motion, and time to radiographic union were similar. These findings suggest that the K-Hammer strategy did not compromise fracture stability or early functional recovery. Flynn criteria were also incorporated to improve comparability with previous supracondylar fracture literature, and most patients in both groups achieved excellent or good outcomes at final follow-up.

The K-Hammer technique may be operator-sensitive, particularly during early adoption. Successful implementation likely requires familiarity with pediatric medial elbow anatomy, accurate localization of the medial epicondyle, consistent manual protection of the ulnar nerve, and controlled single-pass non-rotational advancement of the K-wire. Because all procedures in the present study were performed by experienced pediatric orthopedic surgeons at a single center, the reproducibility of this approach in lower-volume institutions or among less experienced surgeons remains uncertain. Future prospective multicenter studies should evaluate not only clinical outcomes, but also the learning curve, inter-surgeon variability, and standardized training requirements for safe implementation of the technique.

This study has several limitations. First, it was a single-center retrospective case-control study without randomization, and selection bias cannot be excluded. Second, the two groups were not fully contemporaneous because the K-Hammer technique was introduced later during the study period. Therefore, temporal bias, accumulated surgeon experience, and changes in workflow may have influenced the results. Third, although all procedures were performed by senior pediatric orthopedic surgeons, the limited sample size and low number of primary endpoint events precluded robust surgeon-level clustering or adjusted operator-specific analysis. Fourth, the study compared a combined procedural strategy rather than isolating the independent effect of non-rotational hammer-assisted insertion from elbow extension positioning. Fifth, neurological outcomes were assessed retrospectively from clinical records, and electrophysiological testing was not routinely performed. Therefore, subtle subclinical deficits and precise electrophysiological recovery patterns could not be evaluated. Sixth, fluoroscopy acquisition during medial pin placement was not consistently recorded and therefore could not be analyzed. Finally, follow-up duration varied among patients, and the 6-month minimum follow-up should be considered intermediate rather than definitive long-term assessment.

## 5 Couclusions

In conclusion, In this retrospective case-control study of pediatric extension-type Gartland type III–IV supracondylar humeral fractures, the K-Hammer medial pinning strategy was associated with fewer medial pin attempts and shorter operative time compared with conventional freehand medial pinning. The technique also showed a favorable trend toward a lower rate of iatrogenic ulnar nerve injury without compromising radiographic alignment or functional recovery. However, the difference in nerve injury did not reach conventional statistical significance, and the available data do not establish definitive superiority. Further adequately powered prospective multicenter studies are needed to confirm these findings and evaluate reproducibility across surgeons and institutions.

## Data Availability

The original contributions presented in the study are included in the article/Supplementary Material, further inquiries can be directed to the corresponding author/s.
